# P38 mitogen activated protein kinase inhibitor improves platelet in vitro parameters and in vivo survival in a SCID mouse model of transfusion for platelets stored at cold or temperature cycled conditions for 14 days

**DOI:** 10.1371/journal.pone.0250120

**Published:** 2021-05-11

**Authors:** Andrey Skripchenko, Monique P. Gelderman, Jaroslav G. Vostal

**Affiliations:** Division of Blood Components and Devices, Laboratory of Cellular Hematology, Office of Blood Research and Review, Center for Biologics Evaluation and Research, Food and Drug Administration, Silver Spring, Maryland, United States of America; "INSERM", FRANCE

## Abstract

Platelets for transfusion are stored at room temperature (20–24°C) up to 7 days but decline in biochemical and morphological parameters during storage and can support bacterial proliferation. This decline is reduced with p38MAPK inhibitor, VX-702. Storage of platelets in the cold (4–6°C) can reduce bacterial proliferation but platelets get activated and have reduced circulation when transfused. Thermocycling (cold storage with brief periodic warm ups) reduces some of the effects of cold storage. We evaluated in vitro properties and in vivo circulation in SCID mouse model of human platelet transfusion of platelets stored in cold or thermocycled for 14 days with and without VX-702. Apheresis platelet units (N = 15) were each aliquoted into five storage bags and stored under different conditions: room temperature; cold temperature; thermocycled temperature; cold temperature with VX-702; thermocycled temperature with VX-702. Platelet in vitro parameters were evaluated at 1, 7 and 14 days. On day 14, platelets were infused into SCID mice to assess their retention in circulation by flow cytometry. VX-702 reduced negative platelet parameters associated with cold and thermocycled storage such as an increase in expression of activation markers CD62, CD63 and of phosphatidylserine (marker of apoptosis measured by Annexin binding) and lowered the rise in lactate (marker of increase in anaerobic metabolism). However, VX-702 did not inhibit agonist-induced platelet aggregation indicating that it does not interfere with platelet hemostatic function. In vivo, VX-702 improved initial recovery and area under the curve in circulation of human platelets infused into a mouse model that has been previously validated against a human platelet infusion clinical trial. In conclusion, inhibition of p38MAPK during 14-days platelet storage in cold or thermocycling conditions improved in vitro platelet parameters and platelet circulation in the mouse model indicating that VX-702 may improve cell physiology and clinical performance of human platelets stored in cold conditions.

## Introduction

Platelets for transfusion are generally stored at room temperature with agitation after Murphy and Gardner demonstrated reduced in vivo recovery and survival of cold temperature stored platelets (cold-stored platelets) against room temperature stored platelets (room-stored platelets) [1]. Since their report, platelet storage has moved to room temperature in order to optimize circulation of transfused platelets in patients with hypoproliferative platelet disorders. However, limitations of room-stored platelets have emerged over the years and include proliferation of bacteria during storage if the unit becomes contaminated during collection or processing and development of a storage lesion that leads to declining platelet performance with prolonged storage. In recent years, there has been renewed interest in cold-stored platelets because of potential improvement in control of bacterial proliferation during storage and enhanced hemostatic efficacy in bleeding patients due to platelet activation by cold exposure [2–4]. Historically, cold storage of platelets is limited by regulation to 72 hours in the USA [5], but recent research has suggested that platelets can be stored for extended periods of time at cold temperature [6, 7]. Extended cold storage would increase platelet availability for use in massive transfusion protocols [8] and surgical bleeding applications in both civilian and military settings [9].

Platelets stored at cold temperature for longer than 24 hours show irreversible loss of discoid morphology, decreased response to hypotonic shock and increased intracellular calcium, but exhibit reduced metabolic rates leading to lesser glucose consumption and decreased lactic acid production with better maintenance of pH levels [10]. Cold temperature storage also generates changes in cell surface glycoprotein glycosylation, GPIbα clustering [11, 12] and transforms integrin αIIbβ3 conformation to an activated state [13]. Similarly, cold storage manifests in platelet activation which results in lysosomal and granule release [13] as well as increased microparticle formation [14]. Overall, the cold storage lesion appears to make platelets more procoagulant [15] and likely to be effective when transfused into actively bleeding patients [16] even though their circulation time is reduced [1].

Some aspects of the platelet changes induced by cold temperature can be reduced by storage under temperature cycling conditions which periodically rewarm platelets to 37°C for 1 hour in a 12-hour cycle [17]. Thermocycling conditions for 7 days can improve the platelet in vivo recovery and survival in healthy human volunteers compared to continuous cold temperature storage [18].

The changes in platelets induced by cold temperature storage lesion are similar to the activation induced by prolonged room temperature storage [19] which correlates with activation of p38 mitogen activated protein kinase (p38MAPK) [20]. The p38MAPK is involved in a variety of biological processes such as pro-apoptotic signaling, responses to stress and inflammation, regulation of proliferation, cell survival and differentiation [21]. Inhibition of p38MAPK with the chemical compound VX-702 during 7-day storage of platelets at room temperature resulted in better maintenance of platelet functional, structural, metabolic and mitochondrial parameters [20].

The aim of this study was to compare properties of platelets stored in 100% plasma for extended time at cold temperature or under thermocycling conditions with and without VX-702.

## Materials and methods

### Materials

This study was approved by FDA Institutional Review Board. Apheresis platelets were obtained from the NIH blood bank (Bethesda, MD) under informed consent using a cell separator (Amicus, Fresenius Kabi USA, Lake Zurich, IL). Phosphate buffered saline (PBS), Ethylenediaminetetraacetic acid (EDTA), dimethylsulfoxide (DMSO), double-distilled water, human albumin and heparinized capillary tubes, were purchased from Thermo Fisher Scientific (Waltham, MA). Adenosine diphosphate (ADP) and collagen were acquired from Chronolog Corporation (Havertown, PA). CD61 conjugated with fluorescein isothiocyanate (FITC), CD62P, CD63, CD42b, CD29, and annexin V conjugated with phycoerythrin (PE), mouse IgG_1_ PE and FITC platelet isotype control were procured from Becton Dickinson (BD Biosciences, San Jose, CA). Anti-human CD41 antibodies conjugated with FITC were obtained from Beckman Coulter (Indianapolis, IN). VX-702, was bought from Sellek Chemicals (Houston, TX). Severe Combined Immunodeficient (SCID) mice (4 to 6 weeks old) were obtained from Charles River Laboratories (Wilmington, MA). They were maintained in the pathogen-free animal facility of the Food and Drug Administration (FDA) Center for Biologics Evaluation and Research (CBER; Silver Spring, MD) and acclimated for at least 7 to 10 days before starting the experiments. This study was reviewed and approved by FDA’s White Oak Animal Care and Use Committee (OLAW Assurance D16-00655). All procedures were planned and performed using humane practices in accordance with The Guide for the Care and Use of Laboratory Animals (8th Ed.) and standards for accreditation by AAALAC International.

### Platelet collection and study design

Four to 6 hours after collection of single donor platelets by apheresis, platelets in 100% plasma were aliquoted by 65 mL into CLX storage bags (Haemonetics Corporation, Covina, CA). Aliquot A) was stored at 20–24°C with continuous agitation (room-stored platelets); aliquot B) was incubated at 4–6°C without agitation (cold-stored platelets); aliquot C) platelets were stored in an thermocycling incubator (Panasonic, model MIR-154-PA, Wood Dale, IL) programmed to a cycle for 11 hours at 5°C and 1 hour at 37°C with 5 minutes of agitation during the warm-up period using an indoor plug-in timer (Leviton Manufacturing Co., Inc., Melville, NY) as described earlier (thermocycled-stored platelets) [17]; aliquot D) platelets were stored as aliquot B with addition of 10 μM of VX-702 (cold-stored + VX-702 platelets); aliquot E) platelets were stored as aliquot C with addition of 10 μM of VX-702 (thermocycled-stored + VX-702 platelets). Platelets were held at room temperature for 18–24 hours before all five bags were placed under designated storage conditions. The study was carried out with fifteen independent experiments for in vitro measures and five experiments for recovery and survival in a mouse transfusion model.

### Platelet in vitro parameter measurements

All platelet units were sampled aseptically by syringe (approximate volume of 3 mL) on Days 1, 7 and 14. Platelet concentrations and mean platelet volume (MPV) were measured using a hematology analyzer (Poch-100i, Sysmex America Inc. Lincolnshire, IL).

Platelet extracellular pH was measured using a bench top meter (Orion Star A211, Thermo Fisher Scientific) and pH electrode (Ross Ultra, Thermo Fisher Scientific). Glucose and lactate concentrations as well as partial pressures of carbon dioxide (pCO_2_) and oxygen (pO_2_) were measured using a blood gas analyzer (Stat Profile Prime, Nova Biomedical, Waltham, MA). Bicarbonate concentration was calculated using Henderson-Hasselbalch equation from pH and pCO_2_ (37°C) levels.

The extent of platelets shape change (ESC), hypotonic stress response (HSR) and aggregation were measured turbimetrically using a Chrono-Log Model 700 (Chrono-log Corporation) as previously described [22]. Measurements and data collection for these assays were carried out using computer interface with AGGRO/LINK8 software version 1.2.797. All platelet samples were diluted with cold-stored concurrent plasma to obtain 3.0 x10^8^ platelets/mL. ESC, a quantitative measurement of the percentage of platelets that have a discoid shape capable of transforming to spherical form, was performed using 20 μM ADP and 2 μM EDTA according to the manufacturer’s protocol. HSR, a measurement of platelet ability to recover after exposure to hypotonic environment, was executed using PBS and double-distilled water according to the manufacturer’s protocol. Platelet aggregation was measured using 10 mM of ADP with 10 mg/mL of collagen (Chrono-Log), as final concentrations. Agonists were added in a rapid succession one after the other to the platelet suspensions. Aggregation was expressed relative to a maximum slope and amplitude of the instrument’s internal electronic standard. Area under the curve (AUC) was automatically calculated by the instrument’s software.

Platelet activation was accessed by measuring p-selectin expression (CD62P) and Tetraspanin 30 (Tspan-30, CD63) using FACSCalibur flow cytometer (BD Biosciences). Phosphatidylserine exposure on platelet surface, as apoptotic marker, was measured by annexin V binding. Platelet expression of glycoprotein GPIX and integrin β1 were quantified by binding of CD42a and CD29 antibodies, respectively. Briefly, unfixed freshly collected platelet samples were diluted to 1 x10^6^ platelets/mL with PBS supplemented with 0.1% human albumin and were incubated with CD61-FITC and CD62P-PE or CD63-PE or CD29-PE or CD42a-PE or annexin V at saturating concentrations for 15 min at 22°C immediately after sampling. Platelet isotype controls use and color compensation were performed as described earlier [17]. Platelets were gated by forward and side scattering and binding of CD61. P-selectin and Tspan-30 expression were reported as percent CD62P or CD63 positive platelets, respectively. GPIX and integrin β1 expression were reported as mean fluorescence of binding CD42a and CD29, respectively, and phosphatidylserine exposure was reported as percent annexin V positive platelets.

### Animal studies

On Day 14 of storage, cold-stored and thermocycled-stored platelets from 5 platelet apheresis units were transfused into SCID mice. A single animal represented one experimental unit within the compared groups. For this study we used the following number of animals in each group: cold-stored platelets, N = 18; cold-stored + VX-702 platelets, N = 18; thermocycled-stored platelets, N = 15; thermocycled-stored + VX-702 platelets, N = 15. The total number of animals used in all experiments was N = 66. The sample sizes were determined based on previous published study results which refined the experimental design and to reduce variability between platelet units from different donors and flow cytometry data variability [23]. A block randomization was used to assign mice to specific groups. To minimize potential confounders, a simple randomization was used for platelet administration within the group and between the studied groups. The person who infused the mice was aware of the group allocation, the conduct of the experiment, the outcome of assessment and data analysis. The outcome of assessment and the data analysis was also conducted by another team member. Animals were all female SCID mice (CB17/lcr-PrkdcSCID/lcrCr) and were used in all experiments without any anesthetics or analgesics administration. On the day of infusion, mice were acclimated for 2 hours in the procedure room prior to infusion. For the duration of each experiment, the following were employed: a) environmental stressors were kept to a minimum (ambient light and temperature, low noise), b) mice were monitored for behavioral signs of distress, and c) all mice were humanely euthanized immediately upon completion of each experiment. During all experiments, no unanticipated adverse events occurred. The blood sample collections and recovery of human platelets, defined as the percentage of CD41 positive human platelets in murine blood, were managed as described earlier [17, 23]. Briefly, human platelets were supplemented with prostaglandin E1 (1μg/mL) and centrifuged (1000 x *g*, 20 minutes) to concentrate the platelets followed by adding small volumes of plasma to the pellets. Concentrated human platelet aliquots (approximately 5 × 10^8^ in 100 μL) were injected into the right lateral tail vein within 5 minutes of preparation using a 1cc syringe fitted with a 27-gauge needle. All mice were infused with human platelets only once at the beginning of the experiment. All injected mice were included in the experiment except when an injection was not successful. Murine whole blood samples were collected into heparinized capillary tubes using tail vein nick technique at predetermined time points for 6 hours starting at 4 minutes after infusion. After murine blood collections, samples were incubated with a monoclonal anti-human CD41-FITC–conjugated antibodies for 20 minutes in the dark. The flow cytometry gate was established for human platelets based on forward (FSC) and side (SSC) scatter and was used to capture 25,000 events from collected mouse sample.

### Statistics

Determination of means, standard deviations (SD) and standard errors (SE) of experimental values and Analysis of Variance with repeated measures (ANOVA) were carried out by using standard software (Instat, GraphPad Software, Version 3.10, San Diego, CA). A value of p≤0.001 for ANOVA analysis was considered significant accounting repeated measures for the 16 platelet assays utilized in the study and three testing days [24]. If the ANOVA analysis was significant, statistical differences between paired values of room-stored, cold-stored, thermocycled-stored, cold-stored + VX-702, and thermocycled-stored + VX-702 platelet samples were determined by post Hoc tests with Bonferroni corrections. A p value of <0.05 for post Hoc test was considered as statistically significant. Instat software was also utilized to compare human platelet recovery and survival in the animal model. Paired two-tail t test was used for comparison between test and control (GraphPad InStat). Human platelet survival was expressed as an area under the curve (AUC) and was calculated using the GraphPad Prizm software (GraphPad, version 7.05). Two-tailed p value of ≤0.05 was considered significant in paired tests for recovery and survival of human platelets in SCID mice. Two assumption tests were performed when a p value was calculated: pairing effectiveness and normality test.

## Results

### Platelet concentration, mean platelet volume and functional parameters

**Fig 1:**
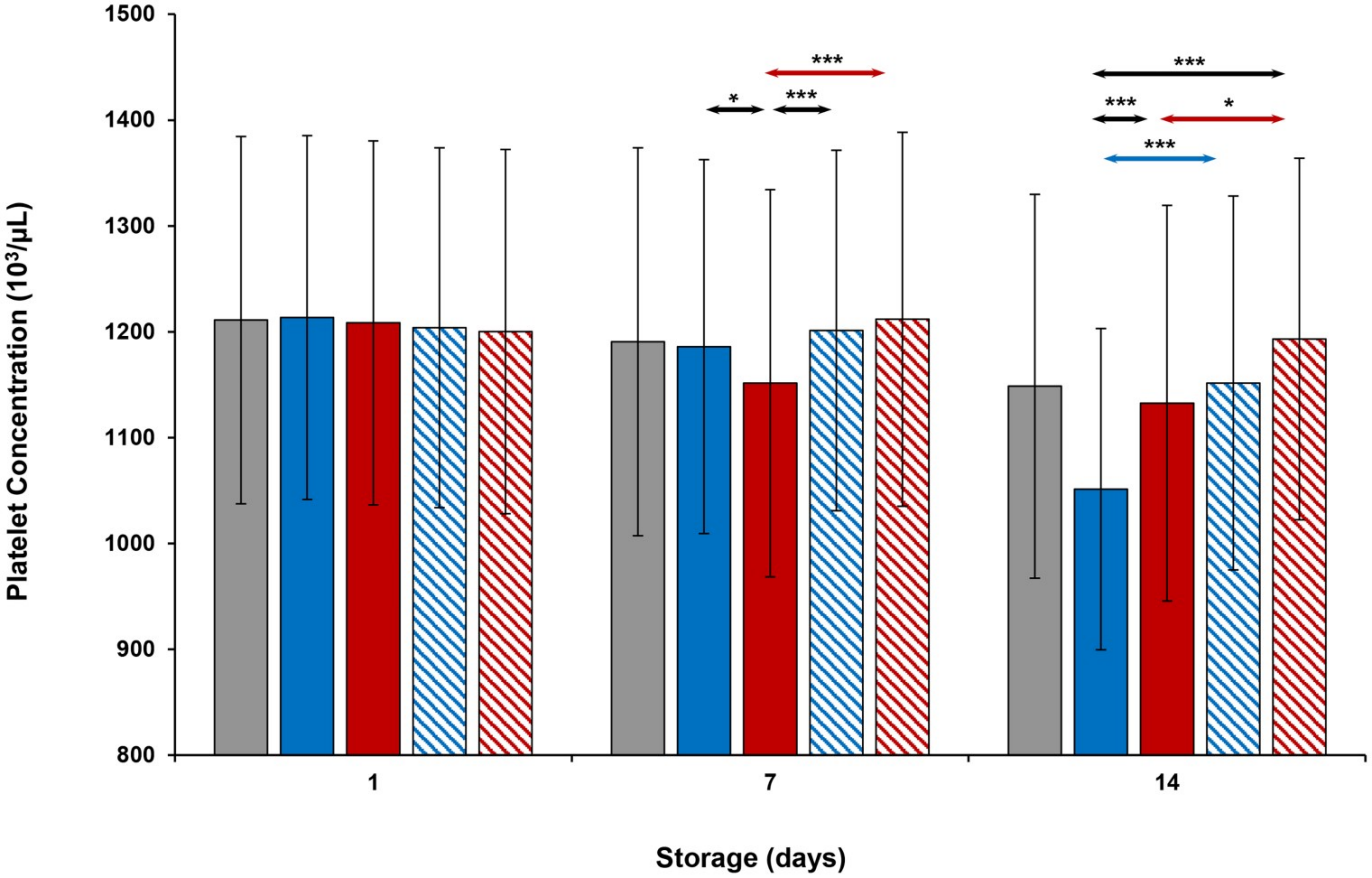
Effects of 14-day storage, temperature and presence of p38MAPK inhibitor on platelet concentration. Grey bar–platelets stored at room temperature; blue solid bar–cold-stored platelets; red solid bar–thermocycled-stored platelets; blue striped bar–cold-stored + VX-702 platelets; red striped bar–thermocycled-stored + VX-702 platelets. Data represents the mean of 15 experiments with one standard deviation. Arrows represent paired statistical differences; *—p<0.05, ***—p<0.001.

**Fig 2:**
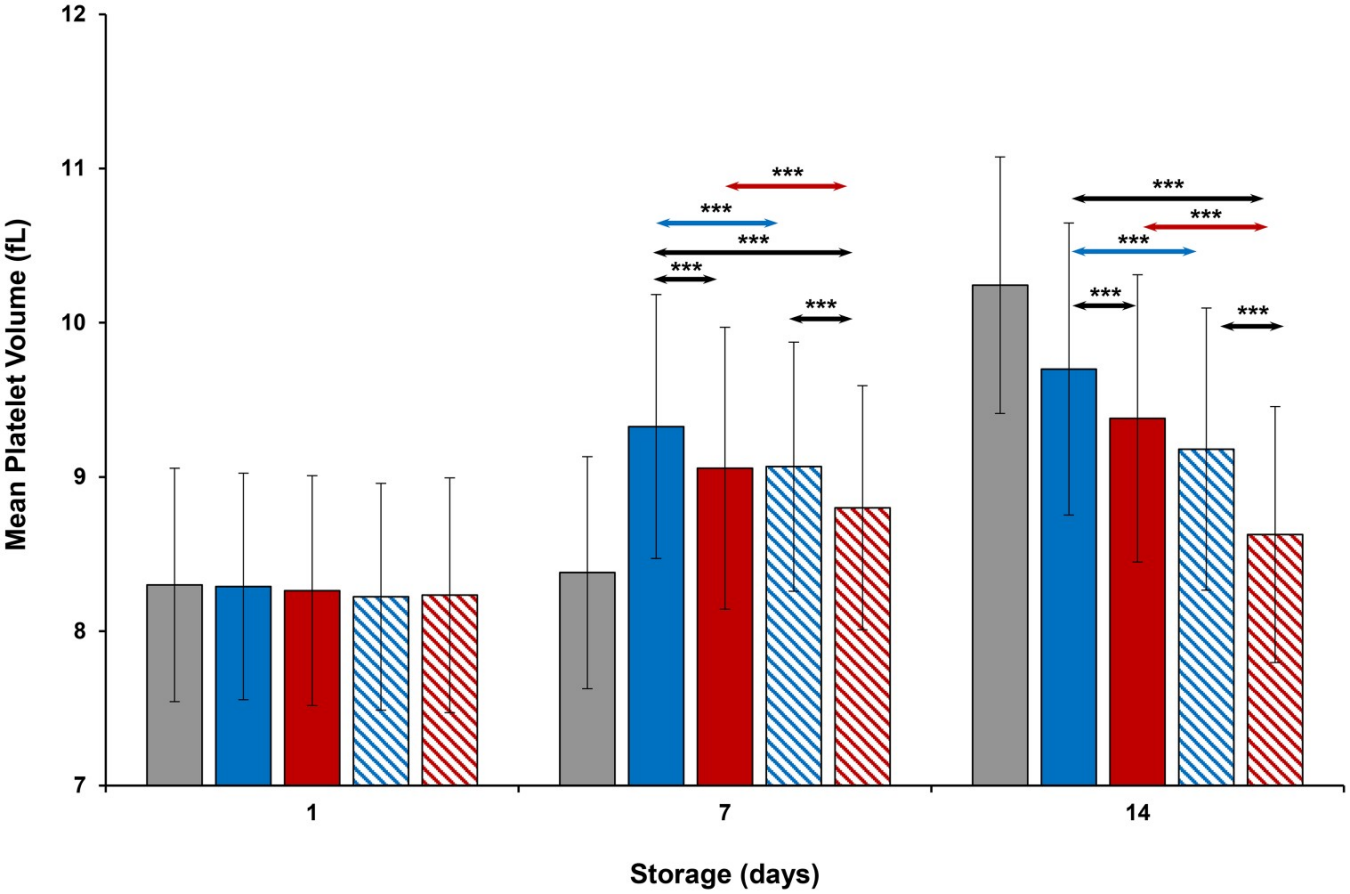
Effects of 14-day storage, temperature and presence of p38MAPK inhibitor on platelet mean volume. Grey bar–platelets stored at room temperature; blue solid bar–cold-stored platelets; red solid bar–thermocycled-stored platelets; blue striped bar–cold-stored + VX-702 platelets; red striped bar–thermocycled-stored + VX-702 platelets. Data represents the mean of 15 experiments with one standard deviation. Arrows represent paired statistical differences; ***—p<0.001.

On Day 1, concentration (cells/μL) and MPV values were similar in all platelets (Figs 1 and 2). On Day 7, thermocycled-stored platelet concentration was less than that of thermocycled-stored + VX-702, cold-stored, and cold-stored + VX-702 platelets. MPV values of cold-stored + VX-702 and thermocycled-stored + VX-702 platelets were less compared to those of cold-stored and thermocycled-stored platelets respectively. MPV values of thermocycled-stored + VX-702 platelets were less than those of cold-stored + VX-702 platelets. On day 14, concentrations of cold-stored and thermocycled-stored platelets were less and MPV values were greater compared to those of cold-stored + VX-702 and thermocycled-stored + VX-702 platelets, respectively. In addition, concentration of cold-stored platelets was less than that of thermocycled-stored and thermocycled-stored + VX-702 platelets (Fig 1).

**Fig 3:**
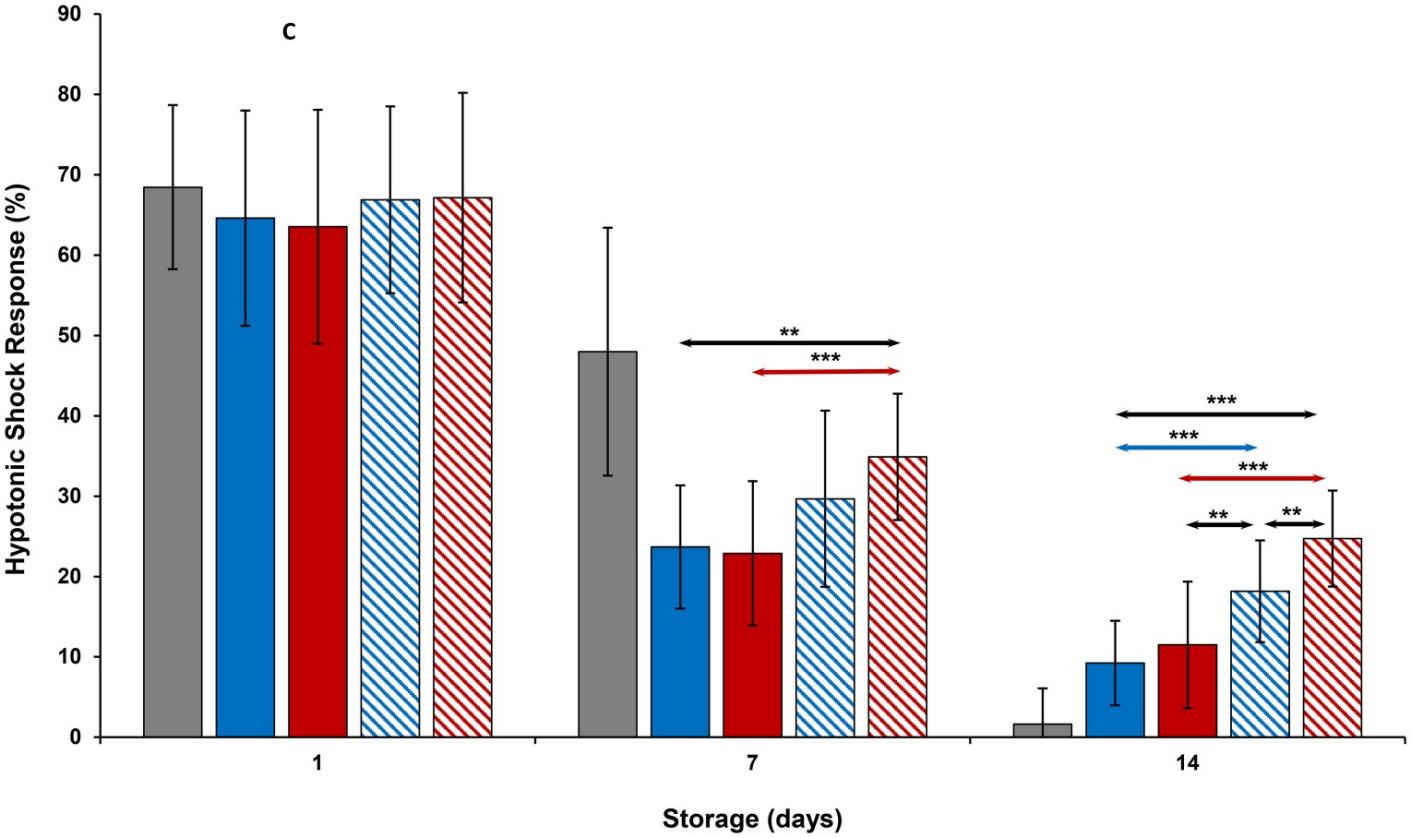
Effects of 14-day storage, temperature and presence of p38MAPK inhibitor on platelet hypotonic shock response. Grey bar–platelets stored at room temperature; blue solid bar–cold-stored platelets; red solid bar–thermocycled-stored platelets; blue striped bar–cold-stored + VX-702 platelets; red striped bar–thermocycled-stored + VX-702 platelets. Data represents the mean of 15 experiments with one standard deviation. Arrows represent paired statistical differences; **—p<0.01, ***—p<0.001.

Results of HSR measurements of all platelets during storage were comparable to each other on Day 1 of storage (Fig 3). On Day 7, HSR of room-stored platelets were greater than those of all other platelets (p<0.001). HSR levels of thermocycled-stored + VX-702 platelets were greater than those of thermocycled-stored and cold-stored platelets. On day 14, cold-stored + VX-702 and thermocycled-stored + VX-702 platelets showed greater values of HSR compared to those of cold-stored and thermocycled-stored platelets, respectively. Moreover, thermocycled-stored + VX-702 platelets had greater values of HSR compared to those of cold-stored + VX-702, cold-stored and room-stored platelets (Fig 3).

Results of ESC measurements are reported in the Tables 1 and 2. On Day 1, all platelets demonstrated similar ESC levels. On Day 7, all platelet ESC levels stored either at cold or thermocycled temperature were less than that of room temperature (p<0.001) but comparable to each other. On Day 14, all ESC measurements were very low, although ESC values of all cold-stored or thermocycled-stored platelets were greater than those of room-stored platelets but similar to each other.

**Table 1:** Values of platelet parameters on Days 1 and 7 of the storage*.

Parameters	Room-stored platelets	Cold-stored platelets	Thermocycled-stored platelets	Cold-stored + VX-702 platelets	Thermocycled-stored + VX-702 platelets
**Aggregation Amplitude (%), Day 1**	92.6±6.1	96.3±9.0	92.4±9.8	94.1±10.1	94.6±9.2
**Aggregation Amplitude (%), Day 7**	84.7±9.3	82.1±16.9	75.7±14.8^d^	75.9±77.9	77.9±17.7
**Aggregation slope (%), Day 1**	129.7±17.0	137.0±22.0	132.5±19.8	130.7±19.6	132.7±20.7
**Aggregation slope (%), Day 7**	87.7±16.6	62.1±13.5	60.1±12.3	57.0±12.2	62.8±13.6
**Aggregation, area under curve, Day 1**	438±33	462±50	441±57	446±61	465±80
**Aggregation, area under curve, Day 7**	390±46	365±83	333±72^d^	328±75	347±85
**ESC (%), Day 1**	31.8±12.3	32.6±11.2	31.7±10.6	31.7±11.9	32.9±11.7
**ESC (%), Day 7**	14.6±6.0	2.2±1.9	2.9±3.0	2.2±2.7	3.2±3.4
**Bicarbonate (mM), Day 1**	23.6±4.8	23.6±4.9	23.4±4.6	23.3±4.7	24.1±4.8
**Bicarbonate (mM), Day 7**	8.2±3.2	15.5±2.8	13.2±3.3^a^	17.0±3.1^d^	15.6±2.9^c^^e^

*—Data presented as mean ± one standard deviation. N = 15;

^a^p<0.001 compared to cold-stored platelets;

^b^p<0.001 compared to cold-stored + VX-702 platelets;

^c^p<0.05 compared to cold-stored + VX-702 platelets;

^d^p<0.05 compared to cold-stored platelets;

^e^p<0.001 compared to thermocycled-stored platelets.

**Table 2:** Values of platelet parameters on Day 14 of the storage*.

Parameters	Room-stored platelets	Cold-stored platelets	Thermocycled-stored platelets	Cold-stored + VX-702 platelets	Thermocycled-stored + VX-702 platelets
**Aggregation Amplitude (%)**	8.3±3.4	70.3±15.4	55.6±19.9^e^	65.4±18.9	60.1±20.4
**Aggregation slope (%)**	26.5±10.7	48.5±12.9	39.3±11.7^e^	44.3±12.8	42.6±14.9
**Aggregation, area under curve**	27±17	286±72	227±92^c^	265±87	250±94
**ESC (%)**	0.0	1.3±1.5	1.6±2.3	1.6±1.9	2.3±2.8
**Bicarbonate (mM)**	0.4±0.1	6.2±2.0	3.2±1.8^a^	8.2±2.3^a^	5.9±2.6^b^^d^

*—Data presented as mean ± one standard deviation. N = 15;

^a^p<0.001 compared to cold-stored platelets;

^b^p<0.001 compared to cold-stored + VX-702 platelets;

^c^p<0.05 compared to cold-stored platelets;

^d^p<0.001 compared to thermocycled-stored platelets;

^e^p<0.01 compared to cold-stored platelets.

Platelet aggregations are presented in the Tables 1 and 2. All platelets demonstrated comparable aggregation amplitude, slope and AUC on Day 1 of storage. On Day 7, all measurements of aggregation slopes for cold-stored or thermocycled-stored platelets were less than those of room-stored platelets but were close to each other. Aggregation amplitudes of thermocycled-stored (p<0.001), thermocycled-stored + VX-702 (p<0.05) and cold-stored (p<0.001) platelets were less than that of room-stored platelets. The aggregation amplitude of cold-stored platelets was comparable to that of room-stored platelets and greater than that of thermocycled-stored platelets (p<0.05). The AUC for aggregation curves followed the difference pattern of aggregation amplitude measurements. On Day 14, all cold-stored or thermocycled-stored platelets exhibited greater aggregation amplitudes, slopes, and AUCs than those of room-stored platelets (p<0.001) but were similar to each other, except that all aggregation measurements of cold-stored platelets were greater than those of thermocycled-stored platelets.

### Metabolic parameters

**Fig 4:**
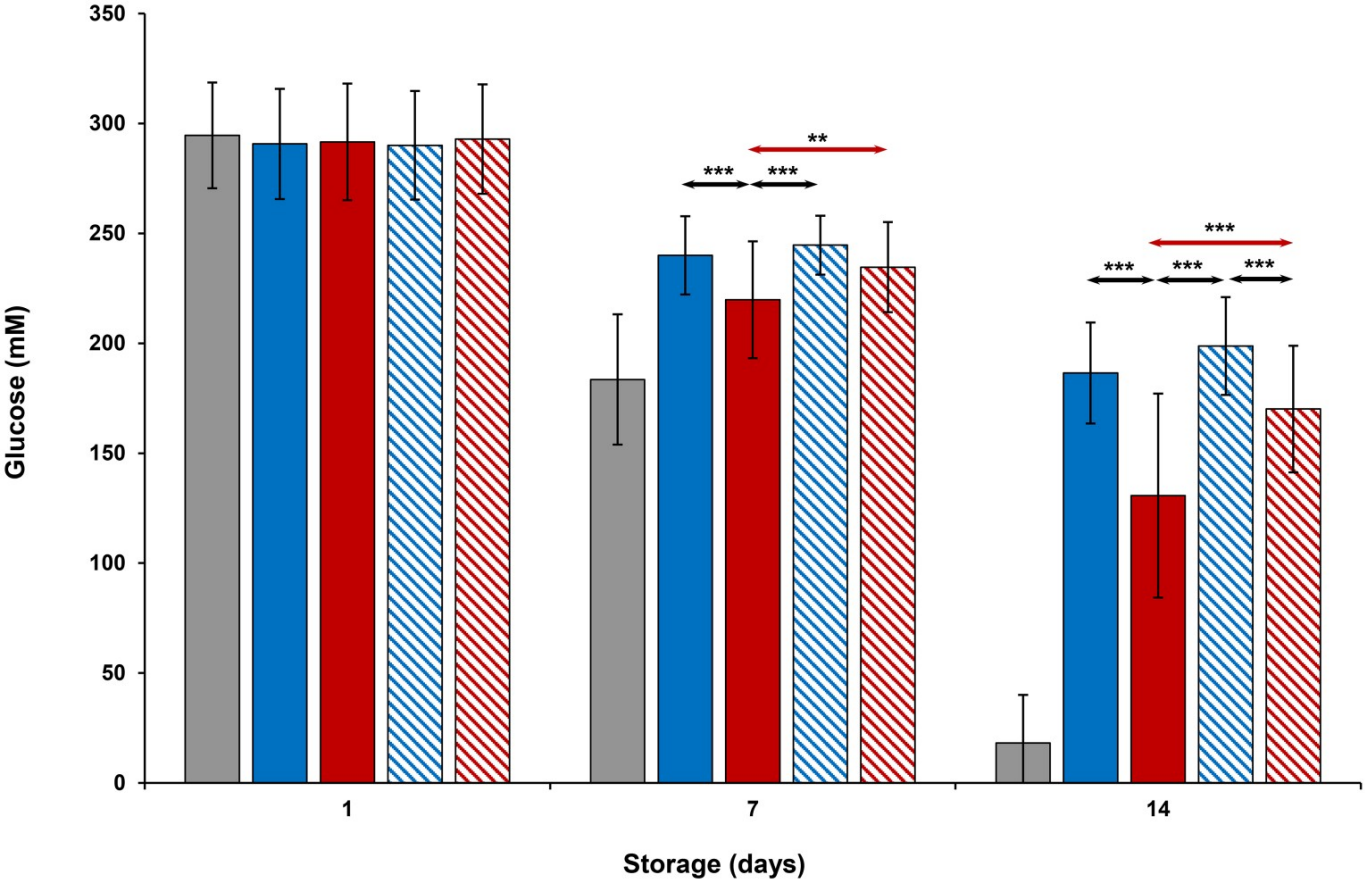
Effects of 14-day storage, temperature and presence of p38MAPK inhibitor on platelet glucose levels. Grey bar–platelets stored at room temperature; blue solid bar–cold-stored platelets; red solid bar–thermocycled-stored platelets; blue striped bar–cold-stored + VX-702 platelets; red striped bar–thermocycled-stored + VX-702 platelets. Data represents the mean of 15 experiments with one standard deviation. Arrows represent paired statistical differences; **—p<0.01, ***—p<0.001.

**Fig 5:**
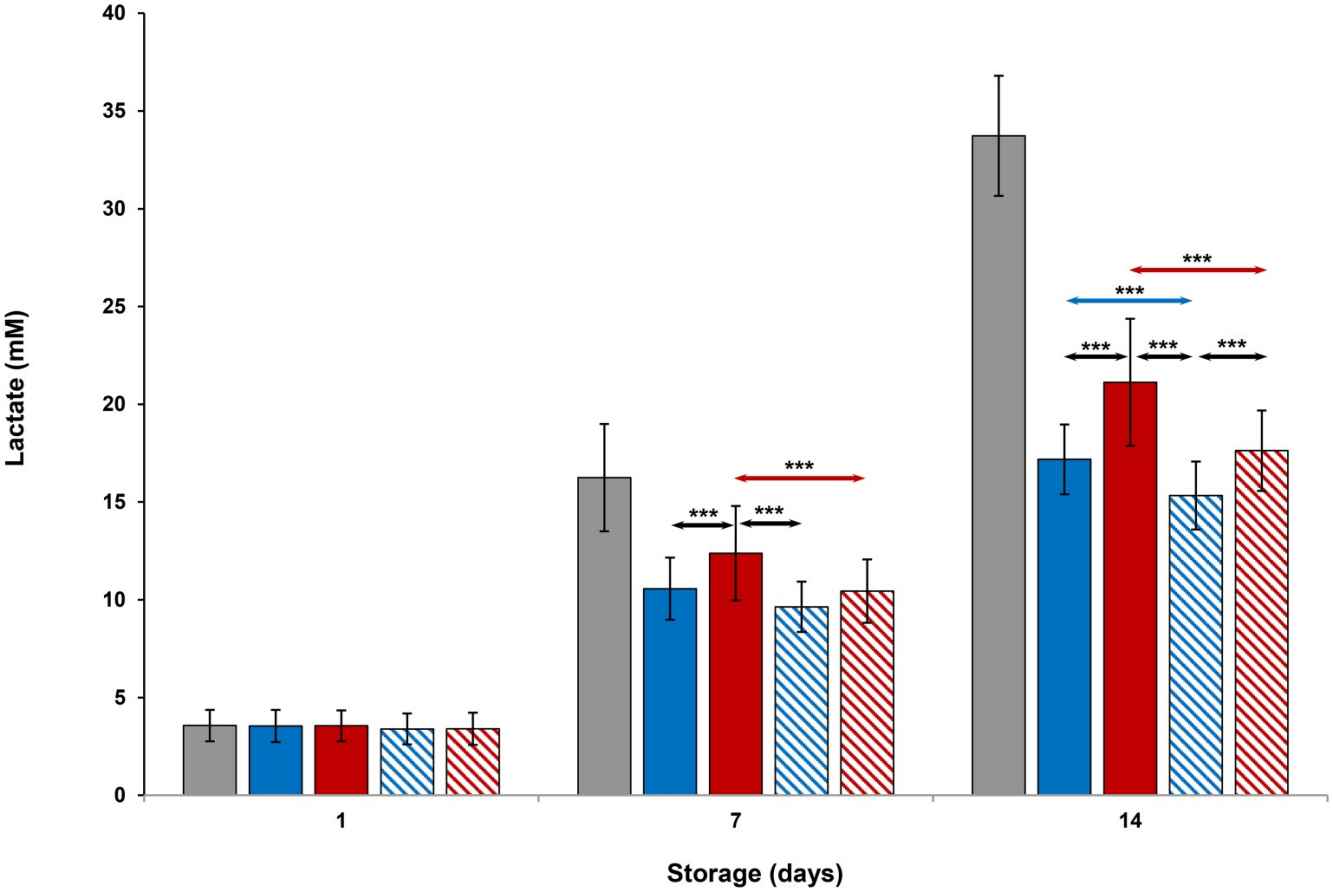
Effects of 14-day storage, temperature and presence of p38MAPK inhibitor on platelet lactate levels. Grey bar–platelets stored at room temperature; blue solid bar–cold-stored platelets; red solid bar–thermocycled-stored platelets; blue striped bar–cold-stored + VX-702 platelets; red striped bar–thermocycled-stored + VX-702 platelets. Data represents the mean of 15 experiments with one standard deviation. Arrows represent paired statistical differences; ***—p<0.001.

**Fig 6:**
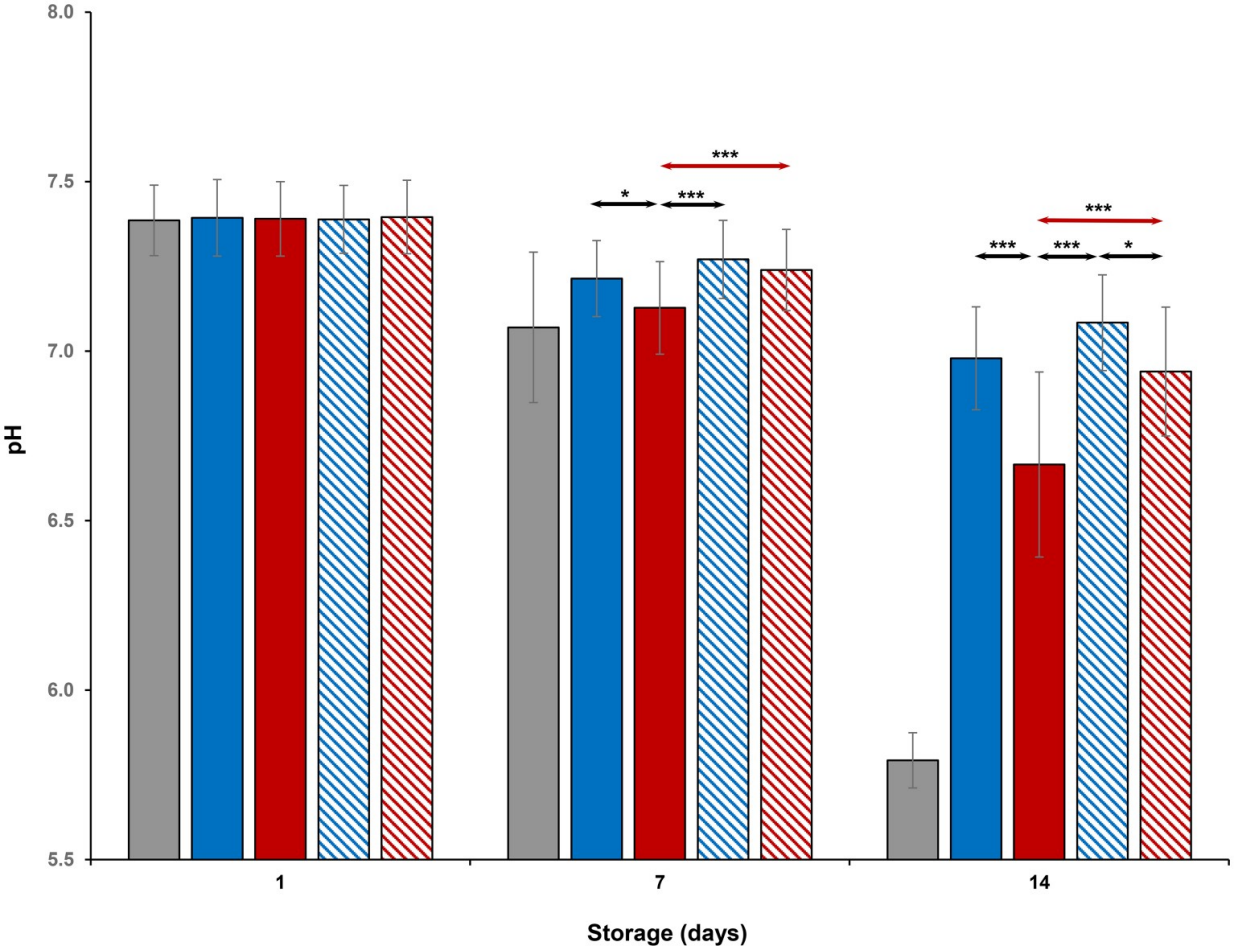
Effects of 14-day storage, temperature and presence of p38MAPK inhibitor on platelet pH levels. Grey bar–platelets stored at room temperature; blue solid bar–cold-stored platelets; red solid bar–thermocycled-stored platelets; blue striped bar–cold-stored + VX-702 platelets; red striped bar–thermocycled-stored + VX-702 platelets. Data represents the mean of 15 experiments with one standard deviation. Arrows represent paired statistical differences; *—p<0.05, ***—p<0.001.

Evaluation of platelet metabolic parameters stored at all conditions are shown in Figs 4–6 and Tables 1 and 2. On Day 1, glucose, lactate and pH levels were comparable to each other (Figs 4–6). Glucose and pH levels of room-stored platelets were less, and lactate levels were greater than those of all cold-stored or thermocycled-stored platelets (p<0.001) on Days 7 and 14. Glucose and pH levels of thermocycled-stored platelets were less and lactate levels were greater compared to those of cold-stored, cold-stored + VX-702 and thermocycled-stored + VX-702 platelets on Days 7 and 14. On Day 14, glucose and pH levels of thermocycled-stored + VX-702 platelets were less and lactate levels were greater than those of cold-stored + VX-702 platelets. Lactate levels of cold-stored + VX-702 platelets were less compared to cold-stored platelets on Day 14 (Figs 4 and 5). The pH levels of cold-stored + VX-702 platelets were greater than those of thermocycled-stored + VX-702 platelets on Day 14 (Fig 6). Bicarbonate levels of all platelets were comparable to each other on Day 1 (Table 1). On Day 7 and 14, bicarbonate levels of -cold-stored + VX-702 and thermocycled-stored + VX-702 platelets were greater than those of cold-stored and thermocycled-stored platelets, respectively (Tables 1 and 2). Bicarbonate levels of cold-stored and cold-stored + VX-702 platelets were greater compared to those of thermocycled-stored and thermocycled-stored + VX-702 platelets, respectively, on Days 7 and 14. Bicarbonate levels of cold-stored + VX-702 platelets were greater than those of thermocycled-stored + VX-702 platelets on Days 7 and 14 (Tables 1 and 2).

### Cell surface parameters

**Fig 7:**
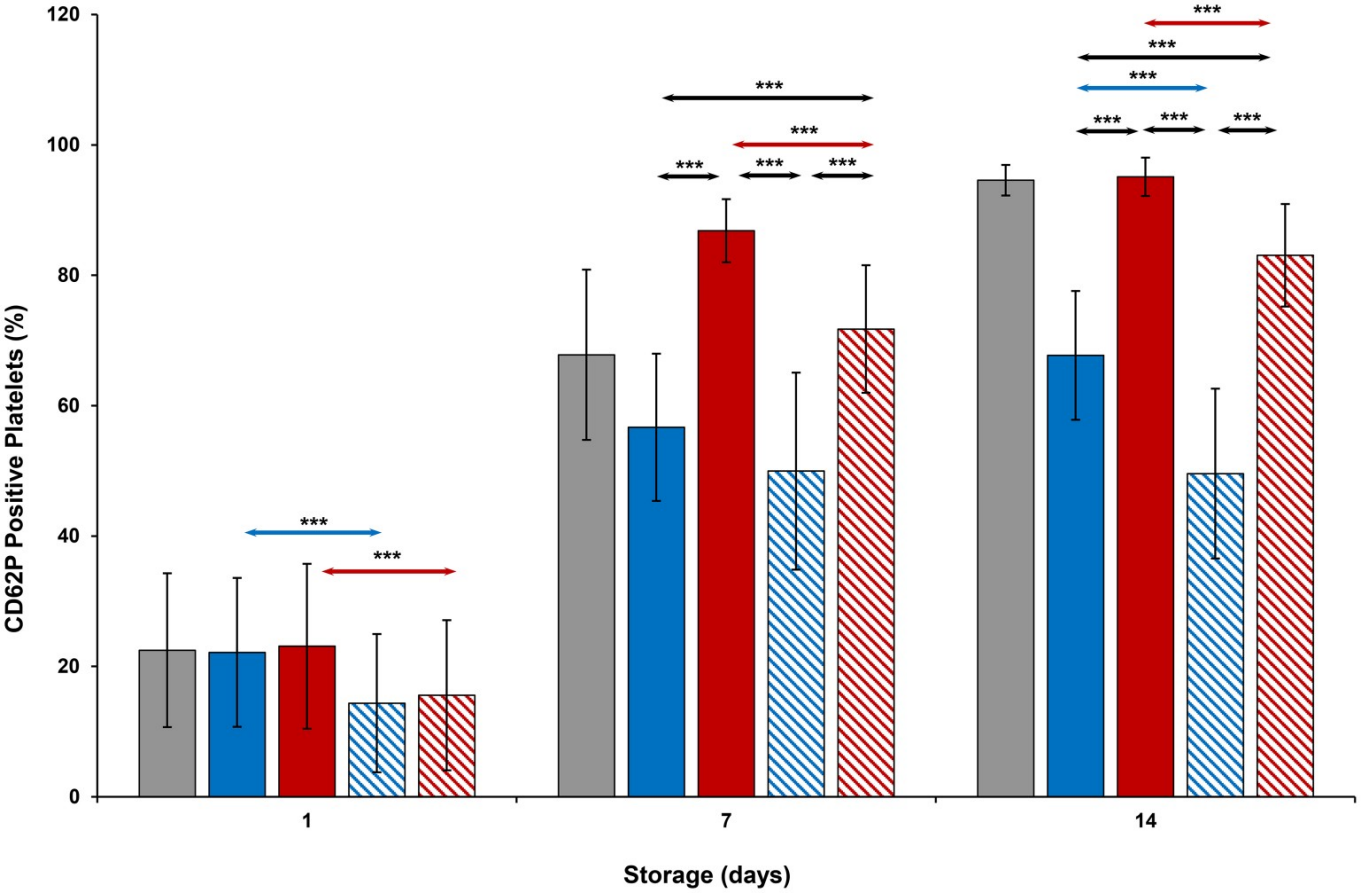
Effects of 14-day storage, temperature and presence of p38MAPK inhibitor on CD62P expression on platelets. Grey bar–platelets stored at room temperature; blue solid bar–cold-stored platelets; red solid bar–thermocycled-stored platelets; blue striped bar–cold-stored + VX-702 platelets; red striped bar–thermocycled-stored + VX-702 platelets. Data represents the mean of 15 experiments with one standard deviation. Arrows represent paired statistical differences; ***—p<0.001.

**Fig 8:**
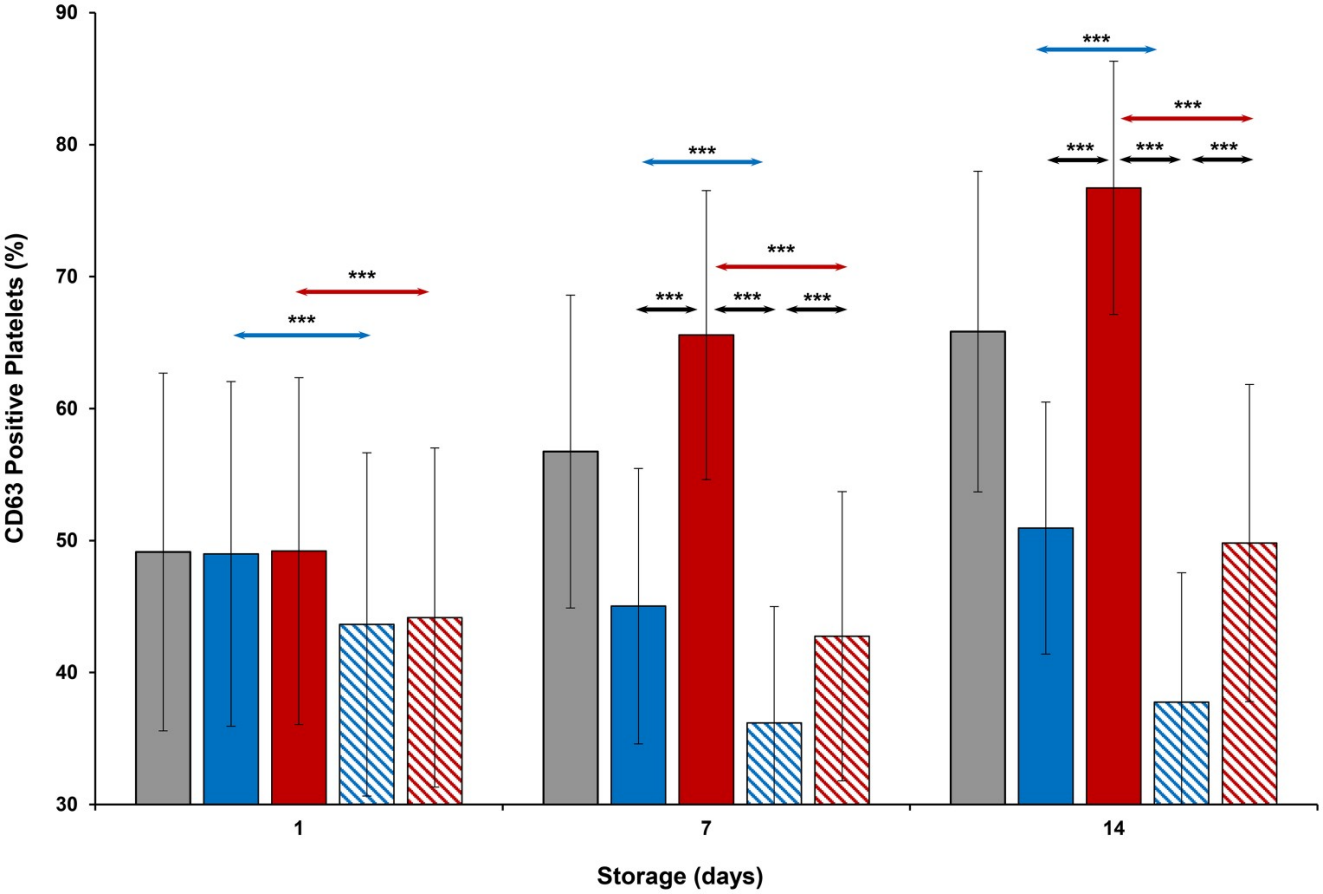
Effects of 14-day storage, temperature and presence of p38MAPK inhibitor on CD63 expression on platelets. Grey bar–platelets stored at room temperature; blue solid bar–cold-stored platelets; red solid bar–thermocycled-stored platelets; blue striped bar–cold-stored + VX-702 platelets; red striped bar–thermocycled-stored + VX-702 platelets. Data represents the mean of 15 experiments with one standard deviation. Arrows represent paired statistical differences; ***—p<0.001.

**Fig 9:**
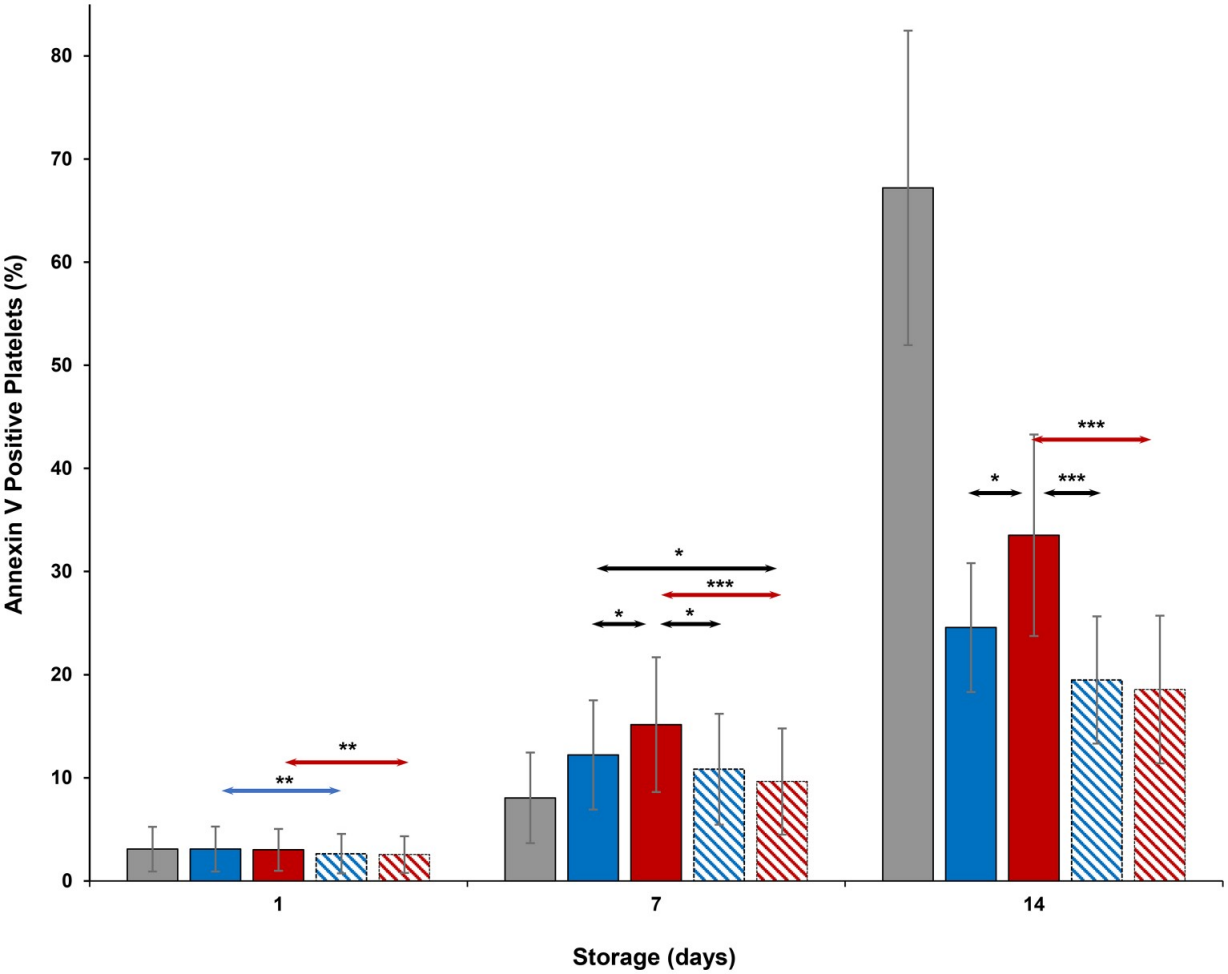
Effects of 14-day storage, temperature and presence of p38MAPK inhibitor on annexin V binding. Grey bar–platelets stored at room temperature; blue solid bar–cold-stored platelets; red solid bar–thermocycled-stored platelets; blue striped bar–cold-stored + VX-702 platelets; red striped bar–thermocycled-stored + VX-702 platelets. Data represents the mean of 15 experiments with one standard deviation. Arrows represent paired statistical differences; *—p<0.05, ***—p<0.001.

CD62P expression (Fig 7) on cold-stored + VX-702 platelets was lower than that of cold-stored platelets on Days 1 and 14, while CD63 expression (Fig 8) of those was lower on Days 1, 7 and 14. At the same time, CD62P and CD 63 expression of thermocycled-stored + VX-702 platelets was lower than that of thermocycled-stored platelets on Days 1, 7 and 14. CD62P and CD 63 expression of cold-stored + VX-702 platelets was lower compared to thermocycled-stored + VX-702 platelets on Days 7 and 14 (Figs 7 and 8). CD62P and CD63 expression of thermocycled-stored platelets was greater compared to those of room-stored (p<0.001), cold-stored and cold-stored + VX-702 platelets on Days 7 and 14, except room-stored platelets on Day 14 when it was comparable for CD62P (Fig 7). Annexin V binding (Fig 9) of thermocycled-stored + VX-702 platelets was lower than that of thermocycled-stored platelets on Days 1, 7 and 14 while it was lower in cold-stored + VX-702 platelets than that of cold-stored platelets only on Day 1. On day 7, annexin V binding of room-stored platelets was lower compared to cold-stored (p<0.001), thermocycled-stored (p<0.001) and cold-stored + VX-702 platelets (p<0.05) but was comparable to that of thermocycled-stored + VX-702 platelets. Annexin V binding on thermocycled-stored platelets was greater than that of cold-stored and cold-stored + VX-702 platelets on Days 7 and 14 (Fig 9).

**Fig 10:**
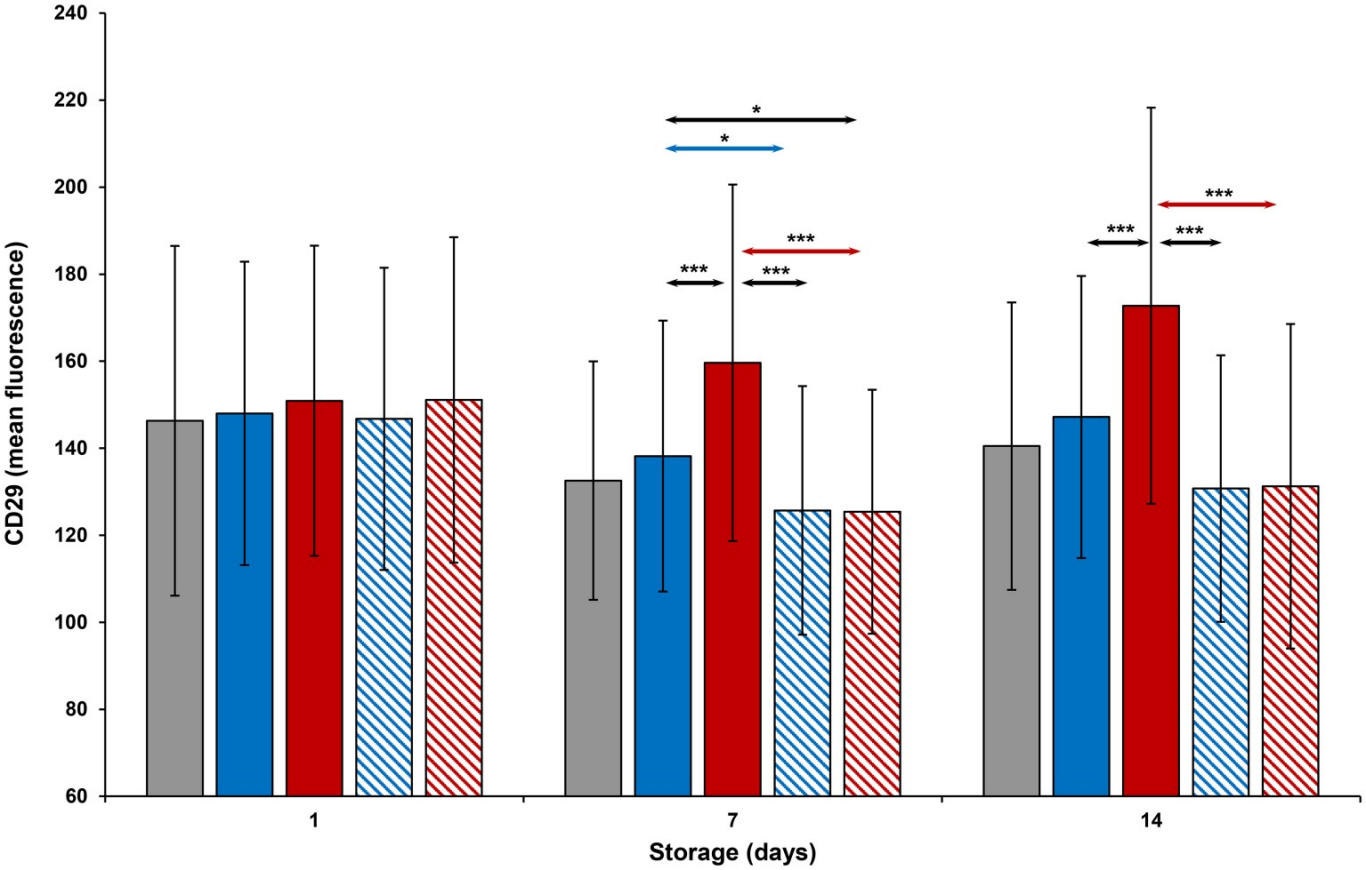
Effects of 14-day storage, temperature and presence of p38MAPK inhibitor on CD29 expression on platelets. Grey bar–platelets stored at room temperature; blue solid bar–cold-stored platelets; red solid bar–thermocycled-stored platelets; blue striped bar–cold-stored + VX-702 platelets; red striped bar–thermocycled-stored + VX-702 platelets. Data represents the mean of 15 experiments with one standard deviation. Arrows represent paired statistical differences; *—p<0.05, ***—p<0.001.

**Fig 11:**
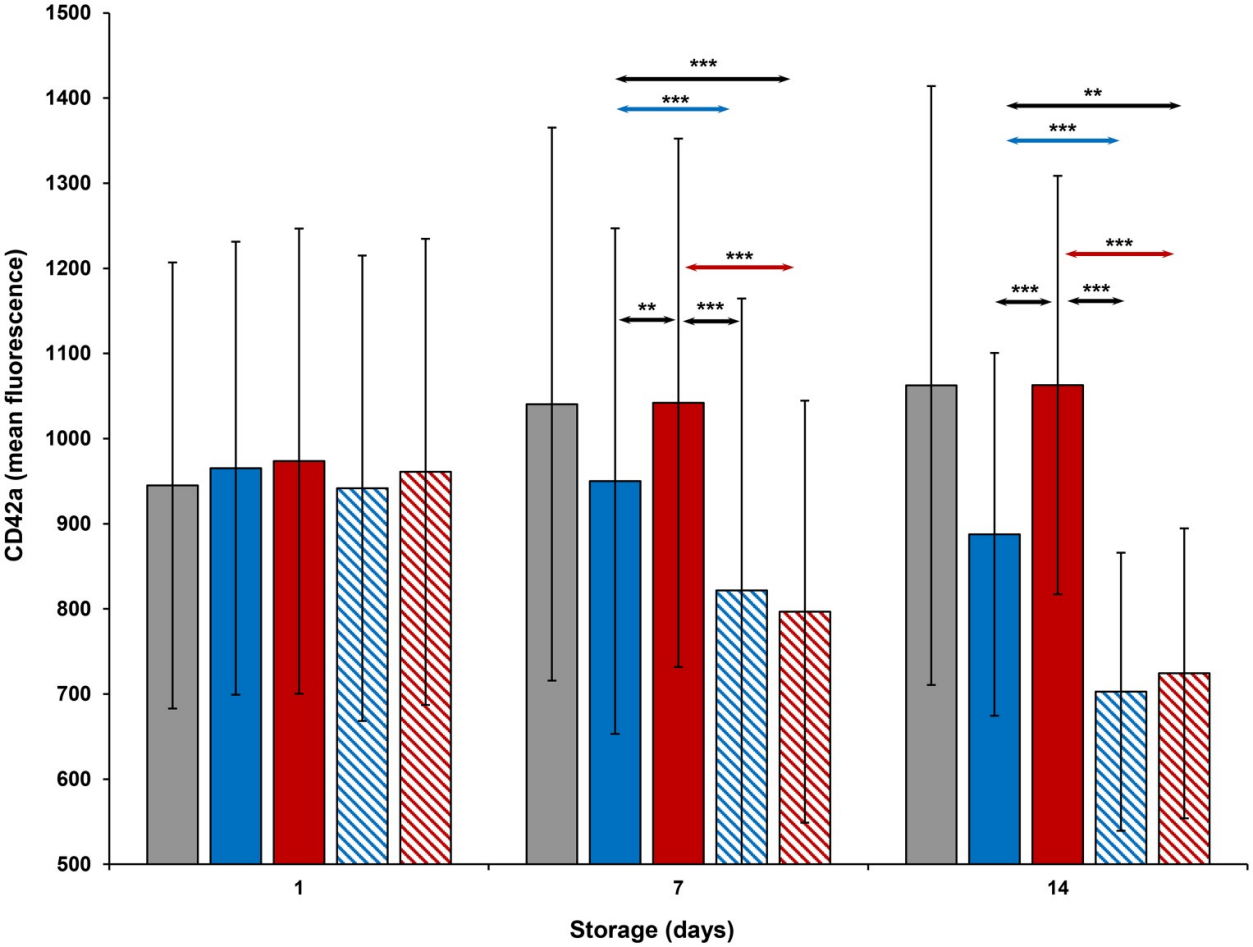
Effects of 14-day storage, temperature and presence of p38MAPK inhibitor on CD42a expression on platelets. Grey bar–platelets stored at room temperature; blue solid bar–cold-stored platelets; red solid bar–thermocycled-stored platelets; blue striped bar–cold-stored + VX-702 platelets; red striped bar–thermocycled-stored + VX-702 platelets. Data represents the mean of 15 experiments with one standard deviation. Arrows represent paired statistical differences; **—p<0.01, ***—p<0.001.

CD29 and CD42a expression (Figs 10 and 11) of all platelets was comparable to each other on Day 1. CD29 expression of thermocycled-stored was greater than that of room-stored (p<0.001), cold-stored and cold-stored + VX-702 platelets on Days 7 and 14. CD29 and CD42a expression of thermocycled-stored + VX-702 and cold-stored + VX-702 platelets was less than that of thermocycled-stored and cold-stored platelets, respectively, on Day 7, while CD42a expression of cold-stored + VX-702 platelets was less compared to that of cold-stored platelets also on Day 14. CD29 expression of cold-stored platelets was greater than that of thermocycled-stored + VX-702 platelets only on Day 7 (Fig 10), while CD42a expression of those were greater on Days 7 and 14. Expression of CD 29 and CD42a was increased on thermocycled-stored platelets compared to cold-stored and cold-stored + VX-702 platelets, on Days 7 and 14 (Figs 10 and 11).

### Animal studies

**Fig 12:**
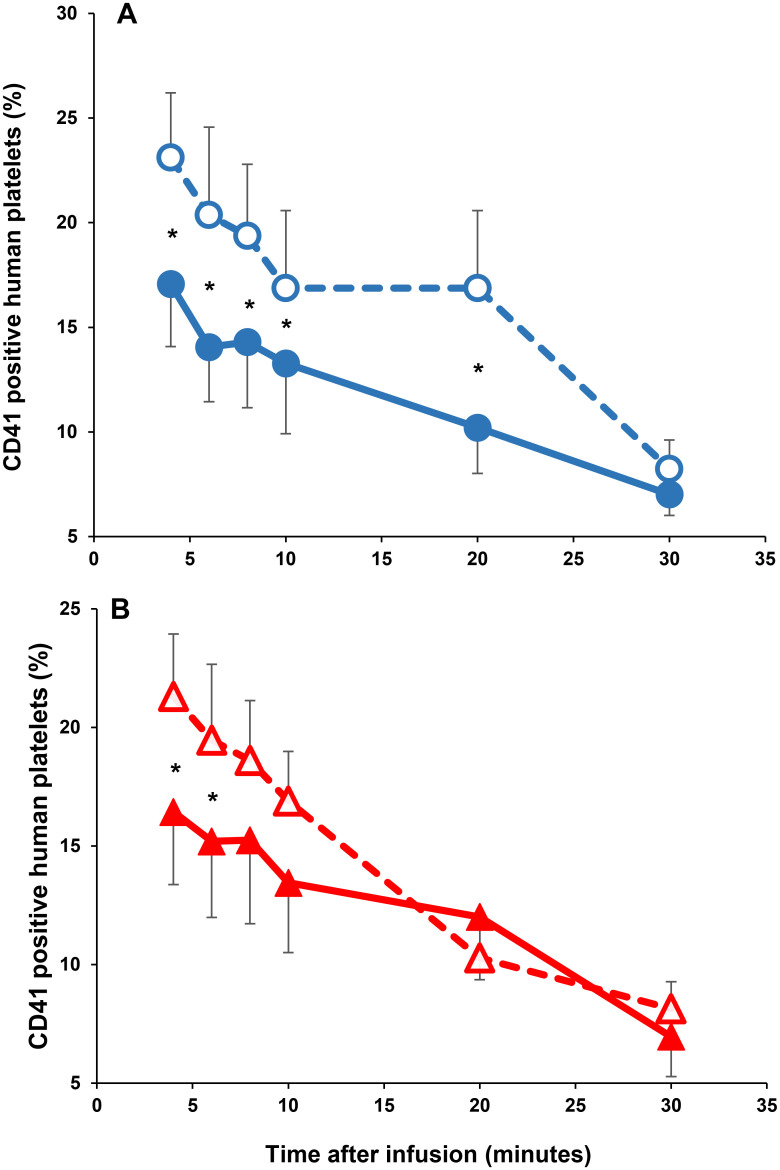
Recovery of human platelets stored for 14 days at cold and thermocycled temperatures. Panel A depicts the recoveries of human platelets stored at cold temperature. Open circles and dashed line represent the recoveries of cold-stored + VX-702 human platelets in SCID mice. Close circles and solid line illustrate the recoveries of cold-stored human platelets in SCID mice; * 4 min p = 0.0001; 6 min p = 0.0126; 8 min p = 0.0127; 10 min p = 0.0053; 20 min p = 0.0177; 30 min p = 0.1402. Panel B provides the recoveries of human platelets stored at thermocycled temperature. Opened triangles and dashed line represent the recoveries of thermocycled-stored + VX-702 human platelets in SCID mice. Closed triangles and solid line reproduce the recoveries of thermocycled-stored human platelets in SCID mice; * 4 min p = 0.0412; 6 min p = 0.01353; 8 min p = 0.1059.

The in vivo recoveries and survivals of human platelets after 14 days of storage (cold-stored, thermocycled-stored, cold-stored + VX-702, and thermocycled-stored + VX-702 platelets) in SCID mice are presented in Fig 12 and Table 3. Recoveries of cold-stored + VX-702 human platelets were greater than those of cold-stored platelets during the first 20 minutes of human platelet circulation in SCID mice (4 min p = 0.0001; 6 min p = 0.0126; 8 min p = 0.0127; 10 min p = 0.0053; 20 min p = 0.0177; 30 min p = 0.1402). Recoveries of thermocycled-stored + VX-702 human platelets were greater than those of thermocycled-stored platelets during the first 6 minutes of human platelet circulation in SCID mice (4 min p = 0.0412; 6 min p = 0.01353; 8 min p = 0.1059). The AUC, which represents availability of human platelets in the murine circulation, was greater for cold-stored + VX-702 platelets (p = 0.03) compared to that of cold-stored platelets but was comparable between thermocycled-stored and thermocycled-stored + VX-702 platelets (p = 0.17).

**Table 3:** Recovery and survival of human platelets following storage at different conditions and transfusion into SCID mice*.

Storage condition	Cold-stored platelets	Cold-stored + VX-702 platelets	Thermocycled-stored platelets	Thermocycled-stored + VX-702 platelets
**Survival (area under the curve)**	1339±85	1605±160^a^	1525±192	1996±144^b^

*—Data presented as mean ± one standard error of mean.

^a^p = 0.03, compared to cold-stored platelets;

^b^p = 0.17, compared to thermocycled-stored platelets.

## Discussion

There are several potential benefits of cold-stored platelets: increased platelet hemostatic efficacy immediately upon transfusion, reduction of bacterial proliferation and preservation of platelet energy stores that would enable extended storage while maintaining hemostatic efficacy. We focused on the latter benefit with the objective to show that platelet storage could be extended to 2 weeks based on maintenance of in vitro responses that have been correlated with preservation of in vivo circulation [25].

A variety of approaches have been tried by others to minimize the platelet activation effects of cold temperature storage to preserve platelet circulation capacity post storage. One approach was based on platelet storage media with second-messenger inhibitors during 9-day of cold temperature storage [26]. These additives helped to prevent platelet count loss, restored HSR and ESC to some extent, maintained surface GPIb and decreased p-selectin expression on platelets. Another storage additive recently studied was N-acetylcysteine which limited platelet activation, stabilized sialidase activity and further reduced metabolic activity [27]. Some physiologic inhibitors, such as prostacyclin and nitric oxide, can also inhibit activation of cold-stored platelets [28]. So far none of these interventions have been accepted into general practice.

Our approach was to inhibit p38MAPK which is a central mediator of multiple signaling pathways in platelets and other cells [21]. In previous studies it has been reported that addition of the inhibitor, VX-702, prevented p38MAPK activation and impeded decrements in many platelet in vitro parameters after exposure to 16°C without agitation for 24 hours [29]. However, VX-702 was less effective when platelets were stored for 7 days at 4°C [30]. One plausible explanation for these findings is that the severity of cold temperature storage lesion at 4°C is more extensive than at 16°C. VX-702 is a competitive inhibitor of ATP binding to the p38MAPK binding site with a half-life in solution between 16 to 20 hours. A single application of 1 μM VX-702 to platelets may not offer enough inhibition for 7 days. An alternative explanation could be that, VX-702, at higher concentration, may bind to off-target kinases or disrupt other signal transducing enzymes that participate in cold induced transformation of platelets. This is less likely since VX-702 has high specificity for inhibition of the p38α isoform over the β, γ and δ p38 isoforms with no appreciable affinity for a panel of other kinases tested [31, 32].

In the present study we stored platelets at cold temperature and thermocycled conditions for 14 days with or without 10 μM VX-702. We showed that cold temperature storage induced loss of platelets during storage, increased activation and reduced platelet responses in in vitro tests after 14 days of storage which corroborates previously published data [26, 27]. The addition of VX-702 to cold temperature and thermocycled storage maintained platelet concentrations up to 14 days of storage, reduced markers of platelet activation such as an increase in MPV [33], p-selectin and Tspan30 expression and limited phosphatidylserine exposure which is considered a consequence of cells going through apoptosis. P-selectin, recognized by CD62P anti-human antibodies on the platelet surface, is a transmembrane protein stored in the α-granules, which becomes translocated into platelet plasma membrane upon activation. P-selectin is localized on the outer platelet membrane and promotes platelet aggregation at areas of vascular injury. Another activation marker, Tspan30, which is located in the lysosomal granules and is also translocated into outer platelet membrane where it is recognized by CD63 anti-human antibodies. These reductions of platelet activation and apoptotic markers when platelets are stored in the cold with the inhibitor VX-702 suggest that in vivo circulation of extended cold storage of platelets could be improved. We previously reported a similar correlation between improved in vitro parameters in platelets stored in thermocycled conditions and better in vivo recovery and circulation of radiolabeled platelets infused into healthy human volunteers [18].

In addition to the general reduction in platelet activation during cold storage, VX-702 also produced some unexpected effects. While VX-702 reduced platelet cold storage induced activation, it did not inhibit agonist induced platelet aggregation. Others have also reported that VX-702 does not impair agonist induced platelet activation during room temperature storage [20, 34]. These results suggest that p38 MAPK is positioned to maintain platelet resting state and its activity is independent of agonist induced signal transduction. Although platelet aggregation in a synergistic response to ADP and collagen was not altered, more work is needed to evaluate response to different agonists (e.g. epinephrine, arachidonic acid etc.) which involve different signaling pathways.

Another unexpected finding was that addition of VX-702 to cold-stored platelets resulted in similar glucose levels, compared to cold-stored platelets, but generated lower lactate levels which suggest that VX-702 promotes alternate glucose utilization pathways. Besides conversion of glucose to lactic acid by anaerobic metabolism platelets can utilize the pentose phosphate pathway, even when glucose utilization is unchanged to generate more reducing power in the form of NADPH rather than lactic acid [35–37]. Alternatively, lower lactate levels in presence of VX-702 may be explained by better maintenance of aerobic respiration, where mitochondria play a pivotal role. A better preservation of mitochondrial function at cold temperature compared to room temperature stored platelets has been previously reported [38] and VX-702 may potentiate this further. More experimental data are needed to further rectify the influence of VX-702 on platelet metabolism.

Greater protection of mitochondrial function is associated with higher quality of platelet function, particularly with improved HSR [39]. In our experiments, addition of VX-702 resulted in increased HSR levels of cold-stored and thermocycled-stored platelets. After one week of storage, room-stored platelets maintained HSR levels better than any cold conditions, as has been described in the literature [14, 17, 29, 30, 40]. However, after two weeks of storage, HSR levels were better preserved during storage at cold and thermocycled temperatures than at room temperature and this was further improved in presence of VX-702. This effect on HSR may be due to reduced platelet metabolism in presence of the inhibitor and preservation of glucose along with the possibility of more efficient metabolism to maintain energy stores [37] and platelet membrane integrity which may also lead to better survival in the circulation after transfusion [41].

Membrane integrity depends on certain levels of platelet cell surface proteins expression and is likely to contribute to clearance of platelets from circulation. Glycoprotein IX, which was measured by binding of anti-human antibodies to CD42a, is essential protein on the platelet membrane surface which forms noncovalent complex with glycoprotein 1bα (GpIbα). This complex is the receptor for von Willebrand factor and is important in platelet adhesion to injured blood vessels. GPIX had stable expression during cold temperature storage but was lower than on room-stored and thermocycled-stored platelets, which substantiated previous data [13]. Signaling through GPIb-GPIX has been associated with accelerated clearance of platelets from the circulation [42]. In one proposed model, cold temperature induces loss of platelet surface GPIbα but also increase binding of vWF to GPIb-GPIX. This, in conjunction with shear stress, may cause unfolding of mechanosensory domain and initiate intracellular signaling that leads to platelet desialylation and phosphatidylserine exposure. These changes were associated with fast platelet clearance from circulation in a mouse model [43–45]. It has been previously reported that addition VX-702 to platelets stored at cold temperature for 24-hours restored GPIbα shedding to the levels found on room-stored platelets [29]. Our current study demonstrates that VX-702 addition decreases GPIX expression on cold-stored and thermocycled-stored platelets which may lead to diminished signaling through GPIb-GPIX and preserve platelet circulation after transfusion. Addition of VX-702 to cold-stored and thermocycled-stored platelets also diminished expression of CD29, a β1 integrin, which was measured by binding of anti-human CD29 antibodies. This integrin forms a complex with integrin α1 and integrin α2 which function as a collagen receptor on platelet surface. In recent studies, β1 integrin has been considered not only as an adhesive molecule but also as a signaling protein which regulates different aspects of platelet functions. For instance, platelet granule (α-granules, dense and lysosomal) secretions were reported upon β1 integrin stimulation [46]. Similarly, VX-702 decreased cold temperature induced p-selectin and Tspan-30 expression, which are released from α-granules and lysosomal granules, respectively. Moreover, it has been known that integrin β1 regulates actin dynamics by actin polymerization after collagen stimulation through β1 integrin activation [47–50]. At the same time, it has been shown that activation of p38MAPK resulted in F-actin reorganization in rat cardiomyocytes as well as actin polymerization in mouse lungs [51, 52]. It is not clear if inhibition of p38MAPK can cause direct regulation of actin polymerization or if it occurs by signaling through β1 integrin or if both pathways are affected simultaneously. More experiments are warranted to better understand interaction of GPIb-GPIX, p-selectin, Tspan30 and different integrins with VX-702 and the impact on platelet survival.

We used a mouse model of platelet transfusion to obtain data on the in vivo recovery and survival of the human cold-stored and thermocycled-stored platelets from this study to determine if VX-702 addition may alleviate the effects of cold temperature storage lesion on platelet circulation. We previously validated this model against a human clinical study of in vivo recovery and survival of thermocycled-stored and cold-stored platelets [53]. Based on this validation, the difference for initial recovery in SCID mice between room-stored and cold-stored platelets correlates with the differences for in vivo recovery of the same types of radiolabeled platelets in healthy human volunteers [18]. For the current study, the animal model revealed that cold-stored and thermocycled-stored platelets stored with VX-702 had an approximately 35% and 30% better initial recovery, respectively, than platelets stored without VX-702. These results suggest that VX-702 will improve recovery of cold-stored and thermocycled-stored platelets in humans although the exact quantitative extent of the improvement is not predictable. A clinical trial of platelets stored at cold temperature in presence of VX-702 is needed to define the exact benefits for platelet circulation.

Our report suggests that inhibition of p38MAPK limits the detrimental effects of platelet extended cold storage as evidenced by in vitro parameters and improvements of platelet circulation in an animal model that has been shown to correlate with circulation performance of cold temperature stored platelets in humans. At the same time inhibition of p38MAPK does not appear to reduce the potential hemostatic advantage of cold-stored platelets. Clinical safety of this drug has been demonstrated in previous clinical trials when it was evaluated as a therapy for immune disorders [54–56] and thus VX-702 would be a suitable candidate for clinical investigations into extension of cold storage of platelets for transfusion. Future work can also focus on combining VX-702 with other pharmacologic treatments that have shown efficacy for preservation of platelets in cold storage.
